# Coordinating Shared Tasks in Human-Robot Collaboration by Commands

**DOI:** 10.3389/frobt.2021.734548

**Published:** 2021-10-19

**Authors:** Alexandre Angleraud, Amir Mehman Sefat, Metodi Netzev, Roel Pieters

**Affiliations:** Cognitive Robotics Group, Faculty of Engineering and Natural Sciences, Tampere University, Tampere, Finland

**Keywords:** collaborative robot (cobot), human-robot interaction, task coordination, knowledge-based, speech recognition

## Abstract

Human-robot collaboration is gaining more and more interest in industrial settings, as collaborative robots are considered safe and robot actions can be programmed easily by, for example, physical interaction. Despite this, robot programming mostly focuses on automated robot motions and interactive tasks or coordination between human and robot still requires additional developments. For example, the selection of which tasks or actions a robot should do next might not be known beforehand or might change at the last moment. Within a human-robot collaborative setting, the coordination of complex shared tasks, is therefore more suited to a human, where a robot would act upon requested commands.In this work we explore the utilization of commands to coordinate a shared task between a human and a robot, in a shared work space. Based on a known set of higher-level actions (e.g., pick-and-placement, hand-over, kitting) and the commands that trigger them, both a speech-based and graphical command-based interface are developed to investigate its use. While speech-based interaction might be more intuitive for coordination, in industrial settings background sounds and noise might hinder its capabilities. The graphical command-based interface circumvents this, while still demonstrating the capabilities of coordination. The developed architecture follows a knowledge-based approach, where the actions available to the robot are checked at runtime whether they suit the task and the current state of the world. Experimental results on industrially relevant assembly, kitting and hand-over tasks in a laboratory setting demonstrate that graphical command-based and speech-based coordination with high-level commands is effective for collaboration between a human and a robot.

## 1 Introduction

Collaborative robots (cobots) are at increasing rate being deployed in industrial environments, sharing tasks and the work space with humans (Villani et al., 2018a). Tasks can be individually configured in a human-robot team setting, where the operator demonstrates task sequences and skills for the robot, and the robot repeats them (Ogenyi et al., 2021). This avoids having to go through a development phase, considerably speeding up integration time. Cobots are crucial for this, as they are small, light-weight and can be safely moved around by a human operator (Kumar et al., 2021).

However, this programming of tasks is typically targeted only for independent robot motions, and task execution usually does not include human-robot interaction or physical collaboration. This implies that programming is still done offline, while the robot and the tasks are being prepared, and the actual execution phase is mostly autonomous execution of the robot. While applications can be found (Sadrfaridpour and Wang, 2017; Johannsmeier and Haddadin, 2017; Darvish et al., 2021) that integrate coordinated actions (e.g., waiting for human input or trigger), still this is pre-programmed and planned to happen at certain specified occurrences. Coordination is thus planned in advance and both agents (i.e., human and robot) act as decided by a fixed protocol. If and when problems occur, or when changes need to be made in the collaboration, the work flow is disrupted and has to be restarted when problems get fixed or when changes are implemented. This limitation affects the natural collaboration and fluency between human and robot (Hoffman, 2019), as no spontaneous actions are allowed besides simply halting the robot and the action plan. While exceptions exist (see e.g., (Darvish et al., 2021), which takes into account last-minute changes of task allocation), task plans are typically short, to avoid a large task plan network that is complex to model and track.

To allow more natural and fluent human-robot interaction, we believe collaboration between human and robot should be coordinated by the human, assisted by the robot and its knowledge and reasoning capabilities. At any given time during the collaboration, the human worker should be able to select suitable actions from the robot to assist the shared task. The robot verifies that the action is suitable and possible, based on its current state of the world and capabilities. Such knowledge is incorporated in a knowledge base that is updated at regular intervals by observations and human instructions. The selection of actions for the robot thus requires human commands to allow for intuitive instructions. Speech and text-based commands are most suitable as, similar to human-human communication (Rocci and Saussure, 2016), semantics can be included.

In this work, we present the developments to allow human coordination in shared human-robot collaborative tasks. The main contributions of this paper are:

• A knowledge-based system architecture that supports reasoning, planning and knowledge integration
• Shared task coordination by human commands, either by a graphical interface or by speech
• Industrially relevant use case scenarios that evaluate the approach

The paper is organized as follows. Section 2 reviews the state of the art in human-robot collaboration and verbal communication in robotics. Section 3 presents the proposed system, with the knowledge and reasoning architecture (Section 3.2) that describes the state of the world, the actors present within it and the capabilities and properties each contain. Then, in Section 3.3 the selection of robot actions is enabled by both a graphical command-based and speech-based user interface that is connected to the knowledge base for reasoning over capabilities and actions. Results of the approach are presented in Section 4 by evaluation of human-robot collaborative tasks inspired from real industrial use cases. Section 5 presents a discussion on the work, including its limitations. Finally, Section 6 concludes the work.

## 2 Related Work

### 2.1 Human-Robot Collaboration

Collaboration between human and robot within industrial environments has received considerable attention in recent years (Villani et al., 2018a; Kumar et al., 2021). Clear distinctions are made between different categories of collaboration, for example, whether tasks and the environment are shared and which agent takes which task (Kolbeinsson et al., 2019). This allocation of tasks requires careful planning and depends on several (in)dependent factors, such as the capabilities of the robot, the difficulty of re-programming and re-configuring the setup, complexity of the task, among many others. Cobots are well suited to be integrated in such environments, due to their light weight, integrated safety functions and human-centered robot programming interfaces (Villani et al., 2018a,b). Industrial integration requires adherence to international standards that assess the safety aspects (i.e., (ISO-10218-1/2:2011, 2011), for industrial robots and systems, and (ISO-15066:2016, 2016), for collaboration) by a formal risk assessment, where, besides the robot itself, additional systems (Halme et al., 2018) can be incorporated to guarantee safety of the human worker. Additional trends in collaboration between human and robot take the fluency of interaction (Hoffman, 2019) or human factors (Chen and Barnes, 2014) into account. This implies that the user experience (Chowdhury et al., 2020) and user acceptance (Müller-Abdelrazeq et al., 2019) is considered by design of the interaction, with suitable technology that improves, instead of hinders, the outcome.

Even though much research and development is ongoing to accelerate the uptake and deployment of collaborative robots, there is no universal solution that fits all. This is perhaps best exemplified by the variety of modalities available for interaction and the magnitude of differences in industrial environments, tasks and contexts. Several different modalities have been utilized for communication, as demonstrated for gestures (Liu and Wang, 2018), augmented and virtual reality (Dianatfar et al., 2021), verbal and non-verbal communication (Mavridis, 2015) and physical interaction (Ogenyi et al., 2021).

The mentioned works on human-robot collaboration demonstrate that communication is crucial in achieving the goals of the interaction. Depending on the modality, this information exchange can take many forms, such is robot goal poses, safety zones, basic commands, task messages, etc. Non-verbal commands, however, typically transmit different information, as compared to verbal commands. Human to human communication, for example, thrives in verbal communication (Rocci and Saussure, 2016), as information can be shared efficiently and with different nuance and meaning. Enriching robots with the capabilities to interpret, understand and react to verbal commands, or even natural language, is, however, still in early stages of development.

### 2.2 Verbal Communication in Robotics

Verbal interaction between humans and robots has seen success in many different cases (Mavridis, 2015; Marin Vargas et al., 2021). Often, literal commands provide the robustness for communication, as the commands are known, and only basic, short sentences are utilized. The step of going beyond literal command-based instructions aims at extending communication to include semantic annotations of commands (Dukes, 2013) or purely natural language (Williams et al., 2015). One advantage of natural language, as compared to literal commands, is the inclusion of semantics, enabling similar expressions in different ways, such that it is most convenient and comfortable for the human. Moreover, higher-level (cognitive) concepts, such as intention, emotion and action, can be (in)directly included in a phrase, as typically present in everyday human language. The extraction of such information for a Natural Language Processing (NLP) system is, however, not an easy feat. State of the art approaches, utilizing deep neural networks (Otter et al., 2022) or other learning based techniques (Sharma and Kaushik, 2017), have shown real-time conversational skills, as, for example by IBM’s Watson (High, 2012) or GPT-3 (Brown et al., 2020).

With respect to robotics, the understanding and acquisition of language can take advantage of the situational nature of a robot, as it is placed in a dedicated environment where tasks and context are known (Taniguchi et al., 2019). Research works have focused on specific contexts for extractions and interpretations of robot instructions, such as manipulation (Misra et al., 2016), grasping (Chen et al., 2021), intention recognition (Mi et al., 2020; Sun et al., 2021) and grounding (Misra et al., 2016; Shridhar et al., 2020; Vanzo et al., 2020). Other approaches interpret natural language through human-robot dialog (Thomason et al., 2015), or utilize additional sensor modalities, such as vision (Sun et al., 2021; Chen et al., 2021). Research has also targeted semantics, both to understand the world and to execute robot actions within it (Ramirez-Amaro et al., 2019). Approaches specific to learning or assigning the semantics of assembly tasks can be found in (Stenmark and Malec, 2014; Savarimuthu et al., 2017).

Most of the presented works consider the tasks as fixed, with little variation in task allocation (Johannsmeier and Haddadin, 2017) or with a low number of total tasks to be executed (Darvish et al., 2021). The reason for this is that with increasing variation in tasks, the task models easily become too large to manage and track. However, when considering Industry 4.0, the trend of smart manufacturing pushes production processes to include wide variations in products, which are to be completed at irregular and unknown time instances. Collaboration between human and robot is suitable to achieve this with higher efficiency than robots (i.e., full automation) or humans (i.e., full manual labour) alone, as it avoids large and complex task plans that include all possible product variations, and avoids large robot programming efforts. The problem then becomes how to command and coordinate robots effectively and efficiently.

In this work, we address the collaboration between human and robot from the point of view of coordination. In order to enable fluent collaboration, human coordination decides when and which robot actions should be executed. This is done by human command phrases (actions and targets), that can be communicated by speech or via a graphical user interface, at any given time during the shared task. Reasoning over the knowledge base that holds an up-to-date world model, then ensures that robot tasks are executed at the correct time (e.g., when the robot is free) and with the correct functionalities (e.g., robot is capable to reach an object). Command phrases, in combination with a dedicated knowledge representation of the world, has the advantage of including semantic annotation to all knowledge, making the system customizable to the user (e.g., by preferred phrases) and to the tasks (e.g., no predefined task plan, but the user decides who does what and when).

## 3 Materials and Methods

The methodology of the proposed approach and its materials are explained by the system architecture and its contents, which includes the knowledge base and reasoning, action planning and the different interaction modalities, i.e., graphical command-based and speech-based.

### 3.1 Terminology

The terminology, used throughout the work is clarified as follows:

**Coordination**-the act of managing actions towards a common goal, while handling problems, conflicts and collaboration.

**Communication**-the exchange of information by different modalities.

**Command**-a word that the robot knows and reacts to.

**Natural Language Processing (NLP)**-refers to the computational approach of analyzing, understanding and manipulating natural language text or speech.

**Automated Speech Recognition (ASR)**-converts spoken language to text.

**Semantic annotation**-is a process of attaching relevant (meta)data.

In context of the human-robot collaborative tasks, our contributions lie in the human coordination of robot actions by utilizing commands, which are known in the system. Automated Speech Recognition (ASR) tools capture the spoken commands and convert them to text. Natural Language Processing (NLP) takes the text and matches them to existing or related phrases by semantic information that is annotated to the contents of the knowledge base. To reduce the complexity in modelling and tracking shared task plans, the tasks commanded to the robot are short (only few actions) and are not integrated in a higher level goal. This implies that a (shared) goal is only taken into account by the human, who coordinates the actions of him/herself and the robot. Nevertheless, as the world and the low-level tasks are represented in an ontology, this allows for their evaluation, before a robot action is executed. Practically, the current state of the world and the task, and the requested commands are evaluated for matching conditions and capabilities. For example, whether the robot can reach a destination or is holding an object for placement.

### 3.2 System Architecture

**FIGURE 1:**
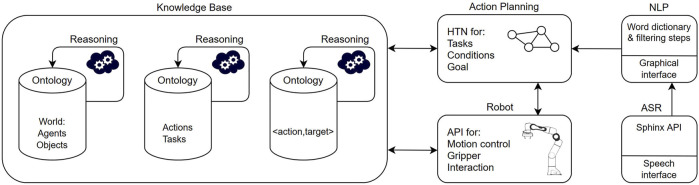
The system architecture is divided in several blocks as follows. The knowledge base (KB) holds all knowledge of the world in form of ontologies, which are updated by reasoning and human input. Action planning generates shared plans in form of hierarchical task networks (HTN), which take input from the KB. Robot capabilities are integrated via a separate robot application programming interface (API) where skills and motion primitives are defined. Automated speech recognition (ASR) and natural language processing (NLP) are separate modules that provide the human input for coordinating the collaborative tasks.

The architecture of our system is based on previous developments on knowledge-based planning for human-robot collaborative tasks (Angleraud et al., 2018). One crucial difference is that no high-level planner is utilized and autonomous robot actions, as inferred from the knowledge base and reasoning, are excluded. Instead, human coordination decides which actions are selected and executed, verified by the reasoning module. The system is depicted in Figure 1 and the individual modules are explained in detail, as follows.

#### 3.2.1 Knowledge Base and Reasoning

Knowledge on the world and its content is represented by ontologies, and referred to as the Knowledge base (KB). As main advantage, ontologies offer a structured description of knowledge, its domain and the relationships that hold between its contents. The KB contains the objects and agents present, and includes relevant information for the tasks and the goals, such as their location, pose, status, etc. Executable actions of the robot, such as end-effector motion, grasping and object placement are expressed as an < action,target > -pair that can be called by the human at any requested instance. The KB and its knowledge representation allow for relationships to be defined between actions, targets and the world state, such that dependencies and conditions can be checked in order to update the KB. Moreover, relationships enable verification of conditions for robot action execution. Reasoning over the KB, therefore, serves two functions:

**World update -** Observations external and internal from robot and the world are utilized to update the KB. For example, the state of the robot, such as end-effector pose, gripper state, and its actions being executed. Moreover, human commands (i.e., < action,target > -pairs) are used to update the KB.

**Action execution** **checks**-Relationships between entries of the KB are checked when robot actions are queried. These pre-conditions verify and enable the execution of robot actions.

Table 1 lists a subset of < action,target > -pairs present in the KB, which is utilized for updating the KB and for coordination of the shared human-robot collaborative tasks.

**TABLE 1:** Coordination of shared tasks is commanded by <action,target>-pairs that specify an action to be executed by the robot, with an accompanying target. Specific details of the architecture are as follows: Pre-conditions-checks whether certain conditions of the world and its content prior to execution are met (e.g., object location, state/capabilities of the robot). Signature-specifies onto which the action/target acts; object, robot and/or human. Semantics-lists the different commands that can be used for triggering the same action/target. Format-describes the underlying knowledge format. Explanation-provides details of the action/target and its specific (sub)tasks.

Action	Pre-conditions	Signature	Semantics	Format	Explanation
moveTo	isWithinReach isReady	Object	Come Go	move action	Move robot end-effector
graspObject	gripperEmpty isReady holdsObject	Object Robot	Pick Take	motion action gripper action	Grasps object
placeObject	isWithinReach isReady	Object Robot	Place Deposit	motion action gripper action	Places object
handOver	isWithinReach isReady humanPresent	Object Robot Human	Give Hand	motion action gripper action	Hand-over object
kitParts	isWithinReach isReady	Object Robot	Kit Stock	motion action gripper action	Pick and place objects
**Target**					
Parts	isWithinReach canBeGrasped	Object Robot Human	Bolt Bolts Tool	3D Pose	Location of parts
Box	isWithinReach isReady isEmpty	Object Robot	Box Kit Container	3D Pose	Location of box
Table	isWithinReach isReady isEmpty	Object Robot	Storage Kit_store Back	3D Pose	Pose on table
Human	isWithinReach isReady humanPresent	Object Human	Here Me	3D Pose	Human hand-over pose

#### 3.2.2 Action Planning

Robot action plans are constructed from mid-level action sequences that execute requested tasks. At a higher level, human coordination guides the collaboration, in order to achieve a shared goal. Action plans are represented by Hierarchical Task Networks (Georgievski and Aiello, 2015), which take input from the KB to generate a plan. On a practical level, this implies that at each action plan node, the state of the robot and the human is checked (e.g., whether the human is active or not, represented by the is Ready state). When the robot is ready, additional pre-conditions of the world are verified that assess whether the actions can be executed (e.g., end-effector pose can be reached: is withinReach, object present: can Be Grasped, gripper empty: gripper Empty; see Table 1, pre-conditions column). When verified correct, the actions are executed. One example explains this planning concept. A robot grasping task is planned as a sequence of actions (i.e., robot motion, gripper motion), where each node in the plan represents the different steps in between the robot actions. At each node, pre-conditions are checked, towards the state of the world and the actions requested, prior to execution. A high level understanding of the shared task is therefore not present in the KB, but only the actions that can be requested from the human. This simplifies the formal planning definition and leaves the high-level coordination towards the shared goal to the human.

#### 3.2.3 Semantic Annotations

Ontologies are well suited to incorporate (semantic) annotations to knowledge. Properties, relations and dependencies can be easily connected to individual entities and link entities together to form (chains of) relationships. Table 1 lists few examples in the pre-conditions column that represent relations and attributes in the world. In regards to the interpretation of semantics towards robot commanding, our system offers the incorporation of semantic annotations, as selected commands can be assigned to address specific actions and targets (see Semantics column in Table 1). Integration of such additional semantics requires the requested commands to be included in the NLP dictionary and the planning domain ontology.

### 3.3 Interaction Modalities

Interaction between human and robot can be divided into modalities utilized for programming robot actions and modalities utilized for task coordination. Physical interaction, such as hand-guiding a robot motion, demonstrates an end-effector pose and is part of a set of robot capabilities developed by the robot manufacturer (i.e., gravity-compensated hand-guiding). Here, we focus on the core interaction modalities of our work, i.e., a graphical command-based interface and a speech-based interface.

#### 3.3.1 Graphical Command Interface

The graphical command-based interface enables a human to instruct actions to the robot, by an < action,target > -pair selected from a graphical user interface (GUI). Based on the current state of the world, a single action can be selected, followed by a suitable target (see Table 1). It has to be noted that the semantics of the actions and the targets are not fixed and can be arbitrarily chosen by the human, by simply changing the terminology in the specific ontology.

#### 3.3.2 Speech Interface

An ASR module enables the shared human-robot collaborative tasks to be coordinated by verbal commands. This essentially relies on the same functionality as the command interface but now, speech has to be interpreted and connected to individual actions and targets to form < action,target > -pairs. While ASR depends on an external software tool, several NLP steps and filters are included to our proposed system. To reduce the complexity of the NLP steps, several additional requirements are set for the acceptance of the command phrases. These are explained as follows.

**Word exclusion**-Common words are removed from a phrase, such as articles (e.g., ‘the’, ‘a’, ‘an’)

**Word limit**-A maximum of two words are accepted for processing.

As general rule, from a command phrase, the NLP system accepts only the words that are defined in the dictionary, and a command phrase should only contain one action and one target. In other cases (e.g., multiple actions/targets or one action/target missing), the command is not accepted. Following, it is checked whether there exist properties of the action and the target, such that a meaningful task can be extracted for the robot. This is done by reasoning and verification over the knowledge base, where all actions and targets are described by suitable properties and relationships.

#### 3.3.3 Knowledge Integration

Integrating new knowledge into the system can be done in various ways, depending on the type of knowledge and its format, as summarized in Table 2. Robot actions to be included are divided in primitive actions, such as single motions or gripper actions, and tasks, which are a list of actions. In both cases the action is demonstrated by the human or programmed in the action library of the robot and linked to the ontology by suitable function call definition. As part of the ontology, conditions should then be defined that will be evaluated before action execution.

**TABLE 2:** Procedure for integrating new knowledge into the system.

Action	Format	Modality	Explanation
Primitive	Robot action	Software integration Python and ontology	Primitive robot actions can be included by function call from ontology to action library
Task	List of robot actions	Software integration Python and ontology	Higher level tasks can be included by defining a list of robot actions
**Target**			
Pose/object	3D pose	Robot hand-guiding	New targets are defined by hand-guiding the robot to a desired pose. This target is then recorded in the ontology
**Other**			
Reasoning rule	SWRL	Software integration Python and ontology	New reasoning rules are defined in the SWRL language and integrated to update the ontology
Synonym	Words	Ontology population	Synonyms to all actions and targets can be included by creating new ontology instances

Targets, which can be locations in 3D space and target objects to grasp (see Table 1), are defined as 3D poses in the world space, to be utilized for the robot, and defined by either hand-guiding the robot or by hard-coding the pose into the KB. This means objects and robot motion are not predefined, but are taught to the system before the execution of the shared task. Annotations to the target in the ontology can be included to provide additional and sufficient information to the target pose. For example, objects such as boxes, into which objects can be placed, require a pose that denotes the location within the box, instead of the pose of the box itself.

Direct inclusion of alternative words (synonyms) for existing commands in the ontologies can be easily done via an ontology editor, such as Protégé (Musen, 2015). This enables semantics to be added to all knowledge, by taking advantage of the functionalities of the OWL2 language. Similarly, new reasoning rules can be added by defining new rules in the SWRL language^1^.

## 4 Experiments

In this section we present the results of our work, by describing and evaluating two use case scenarios that are representative for industrial human-robot collaboration.

### 4.1 Implementation

**FIGURE 2:**
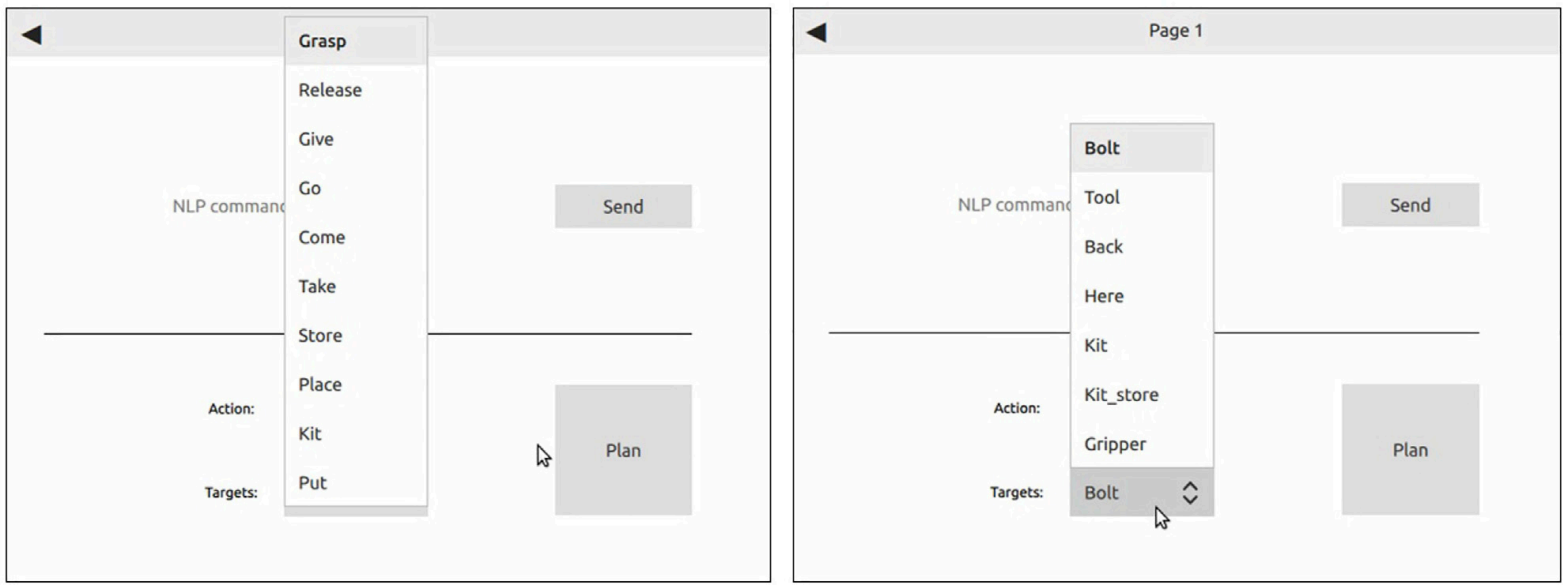
The graphical command interface enables actions and targets to be selected from a drop-down menu. Left: available actions. Right: available targets.

The system architecture and interaction modalities are developed in Python3, utilizing ROS for robot communication and control. The graphical user interface (see Figure 2) is developed in Qt and is launched as single interaction mechanism, enabling also the speech module to pass commands to the system. The Google Speech Recognition engine^2^ enables spoken words to be converted to text. Ontologies are defined using OWL2 standard^3^ with owlready2^4^ and Protégé (Musen, 2015) as ontology editor. Reasoning over the knowledge is done by evaluating rules in the SWRL language1.

Use case scenarios are demonstrated with the Franka Emika Panda^5^ collaborative robot that provides robust motion profiles and control actions for object pick-and-place and hand-over tasks. Industrial parts and tools from a local Diesel engine manufacturer are utilized to demonstrate the capabilities of the proposed system, which includes (collaborative) tasks for the assembly of Diesel engine components and human-robot hand-over tasks for robot assistance. The work environment consists of two tables; one for the robot and parts/tools to be placed, and one for the human operator and the Diesel engine assembly. Both tables are accessible for the human operator and the robot, implying that the whole environment is a shared work space. For the robot, two separate supportive tasks, and thus experiments, are defined:

1. **human-robot hand-overs of parts/tools**-enables the robot to act as a support to workers, while they are engaged in a Diesel engine (dis)assembly task. Hand-over actions from robot to human and from human to robot are coordinated with industrial parts, such as bolts and tools. Human actions include assembly operations of parts to a Diesel engine and the handling of tools.
2. **Robot assisted** **kitting**-enables the robot to group individual items into relevant kits. This assists the human operator in a Diesel engine disassembly procedure, for example by collecting and keeping track of all parts. As a separate activity, kitting occurs alongside the human disassembly procedure. Parts to be handled are bolts and hand-tools.

Both tasks are evaluated by the graphical and speech command interface. A video of the experiments can be seen here: https://youtu.be/SzIuLHzLYpA. In addition, the hand-over task is compared to two baseline methods (i.e., strict turn taking and fast robot cycle), from which clear objective metrics can be extracted that assess the fluency of coordination (Hoffman, 2019). The metrics are human idle time (H-IDL), robot idle time (R-IDL), functional delay (F-DEL) and concurrent activity (C-ACT). For all experiments metrics were calculated with a resolution of 15 sec. This was chosen to be coherent with all experiments and their analysis. In practice, these results were obtained from analyzing the videos of the experiments and finding a common resolution between the different robot and human actions. Experiments were repeated five times. The experimental scenarios are as follows.

### 4.2 Use Case Scenario 1: Command-Based Collaboration

To evaluate human-robot collaboration by commands, a scenario is defined where a human operator selects robot actions (in form of < action,target > -pairs) from a graphical user interface (GUI). The list of actions and targets can be selected from a drop-down menu in the GUI, as depicted in Figure 2. Both tasks, i.e., human-robot hand-over and kitting, are demonstrated as follows.

**FIGURE 3:**
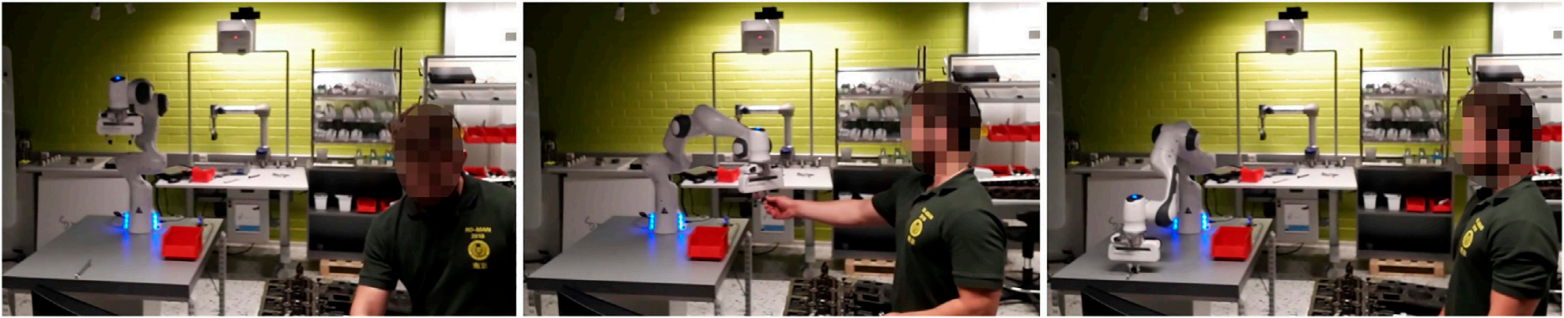
Different stages of the hand-over scenario by graphical command-based coordination. Left: 00:00 - command <give,tool>is send, which instructs the robot to hand-over the wrench from the table to the person. Middle: 01:30 - commands <come,here>and <take,bolt>are used to instruct the robot to receive a bolt from the person. Right: 01:45 - command <give,bolt>is used to instruct the robot to pick up and hand-over a bolt from the table to the person.

**FIGURE 4:**
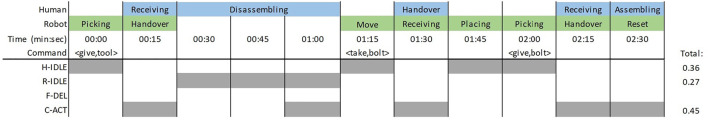
Task assignment chart for hand-over tasks by graphical command-based coordination. The chart depicts the actions of the human (blue) and the robot (green) in the shared collaborative scenario. The fluency metrics indicate a relatively low human and robot idle time (H-IDL and R-IDL) and high concurrent activity (C-ACT). Functional delay (F-DEL) is avoided completely.

Figure 3 depicts different stages of the human-robot hand-over scenario by graphical command-based coordination. Figure 4 depicts a task assignment chart, which visualizes when different agents, i.e., robot or human, are active and with what activity. The commands requested by the human and utilized for robot coordination are depicted as well, and demonstrate the variation in robot actions and how they can be requested. In this case, commands are utilized for object picking and placing (i.e., give and take), robot motion (come) and hand-over tasks from robot to human and human to robot. Parts and locations are described by tool, bolt and here.

**FIGURE 5:**
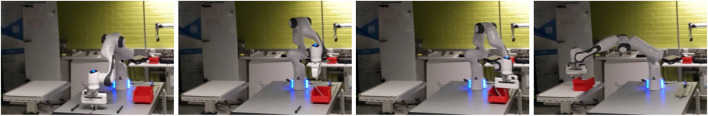
Different stages of the kitting scenario by graphical command-based coordination. Left: 00:00 - commands <pick,tool>and <place,box>are send, which instructs the robot to pick and place the tool from the table to the kit. Second left: 01:00 - command <kit,bolts>is used to instruct the robot to place all bolts from the table into the kit. Second right: 02:00 - command <take,kit>is used to instruct the robot to pick up the kit from the table. Right: 02:15 - command <place,kit_store>is used to instruct the robot to place the kit on the other table.

**FIGURE 6:**
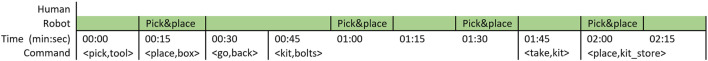
Task assignment chart for the kitting task by graphical command-based coordination. The chart depicts only the actions of the robot (green), as it acts alone.

Figure 5 depicts different stages of the kitting scenario by graphical command-based coordination. Figure 6 depicts a task assignment chart, which visualizes when the robot is active and with what activity. The commands requested by the human and utilized for robot coordination are depicted as well, and demonstrate the variation in robot actions and how they can be requested. In this case, commands are utilized for object picking and placing (pick, place, kit and take) and robot motion (go). Parts and locations are described by tool, box, back, bolts, kit and kit_store.

### 4.3 Use Case Scenario 2: Speech-Based Collaboration

To evaluate human-robot collaboration by speech, a scenario is defined where a human operator requests robot actions (in form of < action,target > -pairs) by speech. A list of actions and targets are available, which are known by the human operator. Again, both tasks, i.e., human-robot hand-over and kitting, are demonstrated as follows.

**FIGURE 7:**
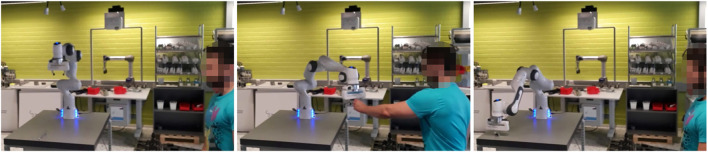
Different stages of the hand-over scenario by speech command-based coordination. Left: 00:00 - command <give,tool>is spoken, which instructs the robot to hand-over the wrench from the table to the person. Middle: 01:30 - commands <come,here>and <take,bolt>are spoken to instruct the robot to receive a bolt from the person. Right: 01:45 - command <give,bolt>is spoken to instruct the robot to pick up and hand-over a bolt from the table to the person.

**FIGURE 8:**
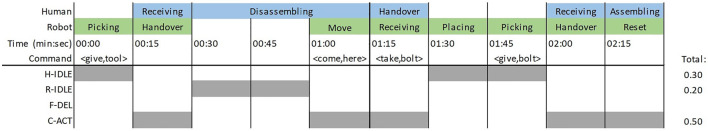
Task assignment chart for hand-over tasks by speech command-based coordination. The chart depicts the actions of the human (blue) and the robot (green) in the shared collaborative scenario. The commands requested by the human to the robot are identical to the graphical command-based scenario. The fluency metrics indicate a relatively low human and robot idle time (H-IDL and R-IDL) and high concurrent activity (C-ACT). Functional delay (F-DEL) is avoided completely.

Figure 7 depicts different stages of the human-robot hand-over scenario by speech-based coordination. Figure 8 depicts a task assignment chart, which visualizes when different agents, i.e., robot or human, are active and with what activity. The commands requested by the human and utilized for robot coordination are depicted as well, and demonstrate the variation in robot actions and how they can be requested. In this case, commands are identical to the graphical command-based hand-over scenario, i.e., object picking and placing (i.e., give and take), robot motion (come) and hand-over tasks from robot to human and human to robot. Parts and locations are again described by tool, bolt and here.

**FIGURE 9:**
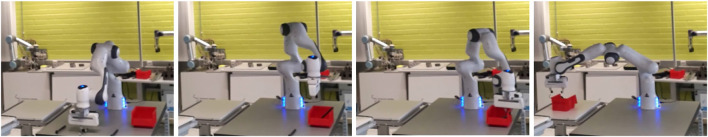
Different stages of the kitting scenario by speech command-based coordination. Left: 00:00 - commands <take,tool>and <deposit,container>are spoken, which instruct the robot to pick and place the tool from the table to the container. Second left: 01:00 - command <stock,bolts>is spoken to instruct the robot to place all bolts from the table into the container. Second right: 02:00 - command <take,container>is spoken to instruct the robot to pick up the kit from the table. Right: 02:15 - command <deposit,storage>is spoken to instruct the robot to place the kit on the other table.

**FIGURE 10:**
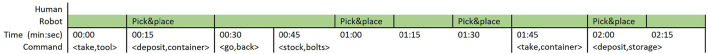
Task assignment chart for kitting by speech coordination. The chart depicts only the actions of the robot (green), as it acts alone. The commands requested by the human to the robot are different from the graphical command-based scenario.

Figure 9 depicts different stages of the human-robot hand-over scenario by speech-based coordination. Figure 10 depicts a task assignment chart, which visualizes when different agents, i.e., robot or human, are active and with what activity. The commands requested by the human and utilized for robot coordination are depicted as well, and demonstrate the variation in robot actions and how they can be requested. In this case, commands are utilized for object picking and placing (take, deposit and stock) and robot motion (go). Parts and locations are described by tool, container, back, bolts, container and storage.

In all cases, robot actions are requested at an instance as decided by the human operator and are executed without major delay, if the robot is not active in other tasks. If the robot is busy, the action is executed as soon as the robot becomes available again.

### 4.4 Baseline Comparison

In order to assess the fluency of collaboration between the human and the robot, resulting from our coordination approach, we devised two baseline approaches as comparison:

1. **Strict turn taking**-each action is immediately followed by the next action of the other teammate, implying that tasks are done sequentially, instead of parallel. Exceptions are the hand-over actions as they require both agents to complete.
2. **Fast robot** **cycle**-each robot action is executed as fast and as soon as possible, irrespective of the actions of the human.

**FIGURE 11:**
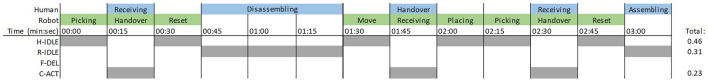
Task assignment chart for the baseline approach of strict turn taking, where each action is immediately followed by the next action of the other teammate. The fluency metrics indicate a relatively high human and robot idle time (H-IDL and R-IDL) and low concurrent activity (C-ACT). Functional delay (F-DEL) is avoided completely.

**FIGURE 12:**
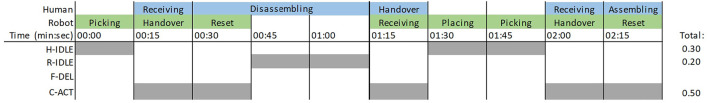
Task assignment chart for the baseline approach of fast robot cycle, where each robot action is executed as fast and as soon as possible. The fluency metrics indicate a relatively low human and robot idle time (H-IDL and R-IDL) and high concurrent activity (C-ACT). Functional delay (F-DEL) is avoided completely.

In both baseline approaches the actions of the robot are not commanded by a human, but executed according to a predefined protocol, as could be found in a factory automation setting. From the objective fluency metrics the following conclusions can be drawn. In strict turn taking (see Figure 11) the human idle time (H-IDL) is relatively high (0.46 or almost half of the time), as concurrent activity (C-ACT) is avoided. Exceptions are the handover tasks that require both agents to collaborate. Similarly, the robot idle time (R-IDL) is also relatively high (0.31 or almost a third of the time), due to the similar reason. The functional delay (F-DEL), however, is avoided, as the agents are never waiting for the completion of each other’s action and are never idle at the same time. In the fast robot cycle approach (see Figure 12) the idling time of both the human (H-IDL, 0.30 or a almost a third of the time) and the robot (R-IDL, 0.20 or one fifth of the time) are low, indicating an efficient utilization of resources. Moreover, the concurrent activity (C-ACT) is high (0.5 or half of the time). The functional delay (F-DEL) is, again, avoided. Following, we compare the baseline approaches to the proposed coordination approaches.

## 5 Discussion

Based on the experiments presented in Section 4, here we discuss and compare their outcome, and present limitations and future work.

### 5.1 Comparison to the Baselines

Human-robot collaboration fluency can be compared in detail according to the metrics of human and robot idle time (H-IDL and R-IDL), functional delay (F-DEL) and concurrent activity (C-ACT). In all cases functional delay is avoided, as the agents are never waiting for the completion of each other’s action and are never idle at the same time. In both command-based approaches the fluency metrics are roughly the same, as the commands are requested at similar time instances during the collaborative task. This indicates a relatively high rate of concurrent activity (C-ACT, almost half of the time) and a relatively low human and robot idle time (for all cases almost a third of the time or less). Compared to all other approaches (fast robot cycle baseline and both command-based approaches) the strict turn taking baseline has the worst performance, with higher idling times (H-IDL and R-IDL), a lower concurrent activity (C-ACT) and the longest scenario execution time. The baseline approach of fast robot cycle has a very close performance compared to the command-based approaches, with minor differences in when actions are executed. This indicates that the proposed command-based approaches are very efficient as the robot has a high utilization rate. However, the most important benefit, which cannot be measured by the fluency metrics, is not possible for both baseline approaches. That is, the flexibility to coordinate and command the actions of the robot at any time and any rate.

### 5.2 Coordination by Commanding

Coordination by commands gives control to the human operator to direct at his/her level of interaction and pace. This implies that its not determined beforehand which tasks are shared and in what level of interaction, leading to an inherently flexible system that suits a wide variety of collaboration. This level of flexibility is not present in the baseline approaches, which assume a predefined sequence of actions, at a fixed pace. Both coordination scenarios demonstrate fluent collaboration between human and robot that is not predefined by a fixed task sequence. High-level robot tasks that contribute to the shared goal (assembly) are object pick and placement and physical interaction for human-robot hand-overs (haptic cues). During each shared task (2.5 minutes), multiple robot commands are requested, i.e., pick and place, and hand-over actions, all while the human operator is engaged, and not disturbed, in the (dis)assembly procedure.

However, a graphical user interface (GUI), even if it approaches the capabilities of a speech recognition system, can be unsuitable for industrial environments. The main reasons identified for this are as follows. First, a GUI takes attention away from the task and the shared environment. Even though this does not necessarily imply danger, it could halt the work or even lead to a reduction in work quality and efficiency. Second, industrial tasks, such as assembly, often require manual handling or manipulation, which cannot be interrupted at random. Collaborative actions would need to be halted and parts would need to be put down in order to interact with the GUI.

Despite these limitations, supportive functions to the GUI can be included to enhance and simplify the interaction. The selection of tasks can be narrowed down by reasoning assistance on the current state of the world (what robot actions are possible) and the actions commanded by the human (what actions are most likely to be needed). This means that only the actions that are suitable at the current moment are available to command and other actions are removed from the selection list.

### 5.3 Commands Vs Speech

To humans, speech is one of the dominant modalities for direct communication (Rocci and Saussure, 2016). In a collaborative work scenario, where manual tasks are taking most attention, speech can be utilized for directing actions and queries to co-workers and, as demonstrated in this work, to robots. However, in industrial environments background noise is very likely to interfere with the reliable recognition of speech. The graphical command interface is one alternative that can replace the recognition of speech, while still ensuring the same functionality of the system. Unfortunately, and unsurprisingly, the graphical interface is less convenient than speech, as it requires manual operation and takes attention away from the task at hand. As a result, robot commanding by GUI takes more time leading to less efficient operations. A disadvantage of the speech interface is it reliability in recognizing correct speech commands and connecting them to the correct action or target phrase. Speech can be misinterpreted and strict guidelines need to be in place that specify how phrases are verbalized. This issue is not present in the GUI interface, as only existing < action,target > -pairs can be selected.

One matter that holds for both interface modalities, is the number and format of commands. As robot skills are plentiful, a limit should be set to how many commands (actions and targets) are available to be executed. In practice, the number of commands to be memorized by the human operator should be limited, as looking up commands from a cheat-sheet has negative effects to a desired fluent collaboration. Likewise, scrolling through a long list of commands from a GUI has the same negative effect. Commands should be intuitive, such that they are directly understandable by the human operator, thereby representing their functionality.

### 5.4 Limitations and Future Work

As demonstrated by the use cases, the system is currently limited by the low number of robot actions and their complexity. However, additional actions, such as motion trajectories, advanced controllers and compound actions are readily available for most collaborative robots and can be added to the knowledge base (see Section 3.3.3 and Table 2). The integration of such new actions involves populating the ontology with instances and creating new relationships and conditions between them. Unfortunately, this is still a manual activity requiring core expertise on ontologies and their properties.

Recent and future developments in speech recognition might offer promising solutions to the mentioned problems in noisy environments. Neural networks and the utilization of other bio-signals (Schultz et al., 2017) are being developed with increasing recognition quality for individual words, as well as for natural language. This includes other sensor systems besides standard microphones, such as throat microphones, or neural devices.

Future work will combine computer vision and speech recognition for collaborative tasks. This allows for tasks that are more descriptive and can be better explained than pre-programmed. For example, a human operator could command the robot to hand over a tool with a red handle from a table with multiple colored tools. Such communication is well-suited to human cognitive skills, as it does not require much cognitive effort for object detection and requesting a command. For robots, on the other hand, such knowledge needs to be integrated beforehand by semantic annotation to the ontology and visual processing of camera images. In addition, feedback from speech recognition can make the system more explainable. Whenever the recognition of speech fails, incorrect words are used or an incorrect combination of words, a suitable command returned to the human would help in improving the collaboration. Similar troubleshooting procedures can be utilized for the reasoning over knowledge as well. Finally, future work will focus on user studies to analyse whether the collaboration is fluent and what concepts contribute to this, specifically targeting concepts such as efficiency, commitment and trust (Paliga and Pollak, 2021).

## 6 Conclusion

Coordination of shared tasks between a human and robot requires interaction modalities that are convenient, do not interfere with the task and can be adapted to new or changing situations. As including all possible scenarios and their outcomes into a robot action plan becomes easily intractable, this work enables a human to coordinate when and which robot actions are executed. Directing the robot is achieved by both a graphical user interface and a speech interface that takes known commands, in form of < action,target > -pairs, and transforms them into low-level actions. All information on actions, tasks and the world is stored in a knowledge base, which is utilized to track the actions and check whether selected actions are suitable or possible at the requested instance. The proposed system is evaluated by several industrial use cases, tested in a laboratory environment, where human-robot collaborative tasks require human coordination by command or speech. Results demonstrate that human coordination with simple commands is suitable to achieve and fulfill collaborative tasks in a fluent manner. Compared to the graphical interface, commanding by speech is preferred, as it does not require physical contact and attention stays with the shared task. On the other hand, noise and faulty speech recognition might prove to be problematic in real industrial environments. A thorough evaluation in real industrial environments, with tasks of similar complexity is, therefore, planned as future studies.

## Data Availability

The raw data supporting the conclusions of this article will be made available by the authors, without undue reservation.

## Footnotes

1
https://www.w3.org/Submission/SWRL

2
https://pypi.org/project/SpeechRecognition

3
https://www.w3.org/TR/owl2-syntax

4
https://pypi.org/project/Owlready2

5
https://franka.de
